# ‘Two heads are better than one’—exploring the experiences of Ghanaian communities on the role of patient and public participation in health system improvement

**DOI:** 10.1093/heapol/czae029

**Published:** 2024-04-17

**Authors:** Samuel Egyakwa Ankomah, Adam Fusheini, Sarah Derrett

**Affiliations:** Department of Preventive and Social Medicine, Ngāi Tahu Māori Health Research Unit, Division of Health Sciences, University of Otago, Dunedin, New Zealand; Department of Obstetrics and Gynaecology, Komfo Anokye Teaching Hospital, P.O. Box GP1563, Kumasi, Ghana; Department of Preventive and Social Medicine, University of Otago, P.O. Box 56, Dunedin 9054, New Zealand; Center for Health Literacy and Rural Health Promotion, P.O. Box 1934, Accra, Ghana; Ngāi Tahu Māori Health Research Unit, Division of Health Sciences, University of Otago, P.O. Box 56, Dunedin 9054, New Zealand

**Keywords:** Patient–public participation, community engagement, health system improvement, sub-Saharan Africa, Ghana

## Abstract

Patient and Public Participation (PPP) is key to improving health systems. Yet, studies have shown that PPP implementations across many countries have been largely tokenistic. Particularly, in Ghana, whilst PPP is prioritized in national health policies and legislation, there appears to be little research focused on understanding PPP’s role in health system improvement. The aim of this study, therefore, is to examine how PPP is working across the Ghanaian health system levels, as well as to understand the perspectives and experiences of participants on how PPP contributes to health system improvement. The qualitative study was undertaken in six communities in three districts in the Ashanti region of Ghana. Data were collected from semi-structured individual interviews. The selection of participants was purposive, based on their PPP-related roles. As a result, findings of this study may not reflect the experiences of others who are not directly involved in PPP initiatives. Thirty-five participants, mainly health service users and health professionals, were interviewed. Data were transcribed and analysed descriptively using Braun and Clarke’s (2006) thematic analysis approach. Overall, participants noted PPP implementation was largely limited at higher health system levels (i.e. national, regional and district levels), but was functioning at the community level. PPP also improved access to health services, responsiveness to patient needs, community-health worker relationships, health-seeking behaviours, empowered healthcare users and improved health outcomes. The study, therefore, recommended the need to undertake PPP across all levels of the health system to maximize PPP’s role in health system improvement. Finally, the study suggested prioritizing PPP, especially for resource-poor countries, to complement government’s efforts in improving accessibility of healthcare services to many communities and also provide a more patient-centred healthcare system responsive to patients’ and public needs.

Key messages

- Patient and Public Participation (PPP) improves access to health services and responsiveness to patient needs.
- Prioritizing PPP will likely complement governments’ efforts in improving the accessibility of healthcare services.
- PPP empowers healthcare users and improves health outcomes.
- PPP implementation is largely limited at higher health system levels (i.e. national, regional and district levels).

## Introduction

Traditionally, Patient and Public Participation (PPP) has focused on improving the relationship between health professionals and patients (Coulter and Elwyn, 2002). However, in recent years, efforts have focused on involving patients and communities more comprehensively, including integrating their experiences and decisions in designing service delivery (Crawford *et al*., 2002; Bate and Robert, 2006). This growing interest in PPP may be borne out of respect for public views and the practical advantages identified (Luxford *et al*., 2011; Draper and Rifkin, 2020). Studies have shown that unengaged patients are mostly dissatisfied (Bate and Robert, 2006). Engagement is one way of satisfying patients and the public. However, focusing on patient–public satisfaction alone, without seeking their views or ensuring these views are genuinely considered in decision-making, has also rendered the PPP process tokenistic rather than genuine (Rowe and Frewer, 2004; Maile and Griffiths, 2014; Ocloo and Matthews, 2016).

Effectively functioning PPP is key to improving health systems (Draper and Rifkin, 2020). Spectra of engagement in health systems have been described as ranging from non-participation (where healthcare users are not allowed to participate) to citizen empowerment (where power is redistributed through negotiation between health service users and health professionals) (Arnstein, 1969; Collins and Ison, 2009). A functioning PPP can include Health Committees and Volunteers, strong feedback mechanisms including scorecards, complaints systems among others to ensure the health system responds to the needs of users (Ankomah *et al*., 2021a; 2023; 2024). While studies have confirmed the importance of PPP in improving health outcomes, there is generally a paucity of literature examining the role of PPP on health systems, especially in Africa, despite the WHO emphasizing PPP as one of the nine key mechanisms for strengthening health systems (Afro Who, 2011; Mafuta *et al*., 2016; Liang *et al*., 2018; Draper and Rifkin, 2020; Ankomah *et al*., 2021a).

Many African countries have prioritized PPP in their national health policies (Christopher *et al*., 2011; Mafuta *et al*., 2015; Dougherty *et al*., 2018). For instance, in Ghana, there are major health policies that directly refer to PPP for health system improvement. In Section 3.1.9 of Ghana’s National Health Policy, PPP is recognized as a key strategy to improve the health system:

…. *to involve communities in the design, planning and development of health interventions is key to facilitating the achievement of high levels of commitment, ownership, and empowerment of communities to champion interventions to improve the health system* (Ministry of Health, 2020; pp. 10).

Further, the National Healthcare Quality Strategy acknowledges the participation of patients and communities as one of seven key strategies for quality healthcare improvement in Ghana (Moh, 2016). Similarly, the Community-based Health Planning and Services (CHPS) policy, introduced in 1999, enables PPP by empowering local communities to have greater control over their healthcare activities (Ghana Health Service, 2005; Baatiema *et al*., 2013). Moreover, the Ghana Health Service and Teaching Hospitals Act, ACT 525 (Government of Ghana, 1996) (sections 18, 23 and 31) also recognizes the importance of PPP as it provides for public representation and participation in health system decision-making across the various health system levels. These levels comprise the national, regional, district, sub-district and community levels (Government of Ghana, 1996).

Despite these policies and legislation promoting PPP, more evidence is needed about how PPP is functioning, or on its overall role in improving the Ghanaian health system (Rifkin, 2014, Alhassan *et al*., 2016, Alhassan *et al*., 2019) Existing findings of PPP’s role in health system improvement in Ghana have been based on grey literature and anecdotal evidence with calls for more rigorous research (Siddiqi *et al*., 2009; Alhassan *et al*., 2016; Agyepong *et al*., 2018; Ankomah *et al*., 2021b). Thus, our study examines how PPP is working across the Ghanaian health system levels, as well as understanding the perspectives and experiences of participants about how PPP contributes to health system improvement.

## Material and methods

### Study setting

The study sites were selected on the basis of having good PPP structures, where participants could share their experiences with the functioning of PPP and its role in health system improvement. Two communities with reportedly strong PPP structures (comprising six case sites) were selected from each of the three districts. These districts and specific case sites were selected through a survey among Health Service Administrators in the Ashanti Region of Ghana to identify districts and communities with good PPP. The survey results were complemented by an ‘insider’ knowledge of the authors and a report from the Christian Health Association of Ghana (Chag, 2014).

Thus, the study was conducted in the Ashanti region of Ghana—specifically the Afigya-Kwabre South, Sekyere South and the Asante-Akim North Districts. The selected districts have a combined population of 440 531 distributed over a geographical area of approximately 1991 km^2^ (Afigya-Kwabre South District Assembly, 2018; Mofep, 2020; 2021). Most residents are peasant farmers earning relatively low incomes. They mostly farm subsistence crops such as maize, cassava, plantains, yams, citrus and vegetables (Mofep, 2020). Most residents belong to the Ashanti ethnic group, and Twi is the most spoken language in the three districts. Regarding healthcare delivery, the Ashanti Regional Directorate of Health Services (RDHS) is administratively responsible for supervising healthcare services in the region. The RDHS also supervise the District Directorate of Health Services (DDHS). In each district, the DDHSs are responsible for implementing health policy initiatives at the local level and for supervising healthcare activities in the district and across communities within the district (Aseweh Abor *et al*., 2008; Ghana Statistical Service, 2021).

### Design and sampling

This study used a qualitative research design to investigate people’s descriptions and understandings of how PPP is working across the Ghanaian health system levels, as well as their perspectives and experiences of how PPP contributes to health system improvement (Bradshaw *et al*., 2017). It was part of a broader qualitative study undertaken to understand PPP’s role in Ghana’s health system improvement. At each site, eligible potential participants were purposively selected because of their roles in PPP activities. A total of 35 participants were interviewed from across the various health system levels, including the national (macro/policy level), district and community levels. The national-level participants included representatives from the Ministry of Health. District Directors of Health Service and Health Service Administrators were selected at the district level. Various cadres of health professionals from the district and the communities were selected: Midwives, Community Health Nurses, CHPS Coordinators and Public Health Nurses. Eligible health service users included members of the Community Health Management Committees (CHMC), Assemblymen/Assemblywomen^1^, Community Health Volunteers (CHVs), Traditional leaders (Chiefs/Queen mothers) and residents of the various communities who were selected on the basis of their past or present experience with PPP. Data saturation was achieved, as at some point, participants provided no new information.

### Data collection

The first author conducted in-person semi-structured individual interviews between December 2020 and August 2021. An interview guide was designed to elicit participants’ views, experiences and opinions on the functioning of PPP in Ghana, and its roles in health system improvement. Before the interviews, the study information sheet was provided and discussed with participants, and written consent was obtained. A Twi language translation of the study information sheet and consent form was available for those who could not communicate fluently in English (both English and Twi are acceptable languages of Ghana). Interviews were conducted in convenient locations of participants—mostly in their homes or offices. Average duration of interviews was 45–60 minutes.

### Data analysis

All interviews were audio-recorded and transcribed by the first author. Those conducted in the Twi language were directly transcribed into the English language. Transcripts were de-identified, and all participants were identified by their positions and assigned unique identifiers for analysis (Saunders *et al*., 2007). The research data were analysed and grouped into themes according to Braun and Clarke’s six phases for thematic analysis (Bowen, 2009; Braun and Clarke, 2022). These include: (1) becoming acquainted with the data; (2) generating codes; (3) identifying themes; (4) reviewing the themes; (5) defining and naming themes and (6) writing about the findings (Braun and Clarke, 2006; 2022). Transcripts were coded within NVivo Version 12 Plus (Qsr International, 2022). The process of inductive coding allowed for new and unexpected themes to emerge from the data. In developing the themes for analysis and interpretation, codes were first assigned to sub-themes and then grouped into major themes (Barnett-Page and Thomas, 2009). The process also involved looking for consistent, co-occurring and overarching themes, including identifying different themes and sub-themes of PPP and their inter-relationships (Alhojailan, 2012). The systematic process of collecting and analysing these research data was duly documented. The research also documented the processes and procedures involved in managing and analysing the data (Gillham, 2000; Saunders *et al*., 2007). This included involving the second and third authors verifying the data coding and reviewing interview transcripts to ensure data consistency and questioning the analysis process to assess for bias about any pre-conceived ideas that may be influencing the analysis.

## Results

Overall, across the six case study sites, 35 participants were interviewed. This was made up of traditional leaders (*n* = 3); residents/opinion leaders (*n* = 4); community health nurses/public health officers/midwives (*n* = 5); CHMC and CHVs (*n* = 11); Assemblymen/Assemblywomen (*n* = 3); CHVs only (*n* = 3); Health Administrators (*n* = 2); District Directors of Health Services (*n* = 3); and Representative of MOH (*n* = 1). Most participants were male (70%); 30% were female. All health worker participants were educated up to the tertiary level (32%). However, 17% of the non-health workers (community-level participants) had tertiary education, 11% secondary education, 37% junior high school and 3% reported no formal education. The average age of participants was 50 years (ranging from 30 to 70 years). The average time participants served in a PPP-related role ranged from 2 to 29 years (Table 1).

**Table 1.** Participants’ role, case site (CS) and interview length

	**Afigya-Kwabre District**		**Sekyere-South district**		**Asante-Akim North district**			
**Participant**	**CS**	**CS**	**CS**	**CS**	**CS**	**CS**	**Unclassified location**	**Average interview length**
**Semi-structured individual interviews**								
Traditional leader (Chief/Queen mother)	1	1	1	0	0	0		45–60 min
Resident/Opinion leader	1	0	0	1	1	1		45–60 min
Health professionals (e.g. Community Health Nurses, Public Health Officers, Midwives)	1	0	1	1	1	1		45–60 min
CHMC & CHV (combined role)	2	1	2	2	2	2		45–60 min
Assemblyman	0	2	1	0	0			45–60 min
Community health volunteers only	0		1	0	1	1		35–60 min
Health facility manager/health administrator	0	1	0	0	1	0		60–85 min
District Director of Health Care Services	1		1		1			45–60 min
Representative—Ministry of Health							1	45–60 min
**Total**	**11**		**11**		**12**		**1**	**35**

### Functioning of PPP at the different health system levels

The study found that PPP was implemented differently across the various health system levels. Whilst PPP implementation was expected to be effective across all levels of the health system as required by the Ghanaian law (ACT 525 of Government of Ghana, 1996), the participants indicated PPP implementation has been limited at the higher levels of the health system (national, regional and district levels). PPP was functioning mainly at the community level of the health system. Participants, however, indicated PPP implementation across the national, regional and district levels could have been more effective considering its importance to improving the health system. A District Director of Health Services mentioned:

*It would be good to have the public involved in major health policy decisions right from the Ministry and even the highest decision-making body of Ghana’s Health Service. But I do not think we are there yet* (District Director of Health Service, 1).

A CHMC member also commented:

*Seeking different opinions in decision-making is the best. Most especially when the decision even affects the lives of many people. Our elders say two heads are better than one. For me, that is why the community’s involvement in health must not only exist in this community but at the District or even the Regional levels as well* (CHMC member, CS 1).

A representative from the Ministry of Health observed:

*You see, it will be good to have effective user involvement even in our plans and policy developments right from the Ministry to the community, but that has not happened yet. We see PPP as more of a community activity than a national one. So, for now, such user engagement strategies have been implemented only in the communities* (Representative, MOH).

This finding shows the current focus of PPP in the Ghanaian health system is mainly at the community health system level. Whilst this can improve the health system, we also recognize that the strength of any health system emanates from its various interconnections. Therefore, the suboptimal functioning of PPP across the higher levels of the health system was likely to adversely affect the overall role of PPP in health system improvement.

### Roles of PPP in system improvement

Participants further reported that PPP significantly contributed to health system improvement. Improvements were categorized into six key themes: (1) improved access to healthcare services, including medicines and other diagnostics services; (2) improved responsiveness of the healthcare system to patients’ needs; (3) improved working relationships between health workers and the community members; (4) improved health-seeking behaviours of the public; (5) enhanced community empowerment; and (6) an overall improvement in the quality of healthcare services.

### Improved access to health care services

PPP was reported to improve access to various healthcare services in the communities. For instance, PPP led to the establishment of new community clinics, improvement in drug/medicine availability and other diagnostic services and an increased number of healthcare personnel offering services in the communities.

In four of the six community sites, participants reported that PPP led to either the construction of new community health clinics or the renovation of old buildings for community health services. For example, at Case Site 1, during an implementation of a CHPS programme, the community donated land and worked with the District Health Directorate and the District Assembly Office to construct a new community clinic. Community members worked voluntarily as construction site workers (e.g. manually digging a trench to lay pipes to connect drinkable water to the clinic) and donated various building materials for this project. A member of the CHMC explained how the construction of the clinic had improved access to healthcare services in the community:

*Previously, our people had to travel very far to other communities for health care services. In most cases, because they could not afford the cost of transport, they would stay in their homes and take unprescribed herbs and other medications, which eventually were bad for their health. However, the situation has improved with the clinic, which of course, came up as a result of our efforts with the health authorities. Now, the community has a place for their healthcare treatments* (CHMC member, CS 1).

Apart from the significant improvement in accessing health care facilities, participants also reported that PPP improved access to medicines and other diagnostic services such as laboratory investigations, x-rays and ultrasound scans. For instance, in case site 2, participants mentioned that the community, through their representatives on the health committee, played significant roles in the establishment of x-rays, new laboratory services and other dental diagnostic services. A Health Administrator stated:

*The Chief and the Assemblyman on the Health Committee really initiated these efforts and further contacted some of their indigenes from abroad to come and support. We had two renowned indigenes of this community who are professors outside who came in to support us financially and logistically to establish the x-ray. Another person also bought the biochemistry machine for us to commence those laboratory tests here* (Health Administrator 1).

In addition, participants reported a significant improvement in medicine availability due to PPP activities. Particularly, in case sites 1 and 3, communities mobilized substantial financial resources to purchase drugs for the community clinic. In some instances, members of the CHMC solicited donations of drugs from pharmaceutical companies to improve the drug availability in the community clinics:

*When the clinic started, we did not have any drugs. The community mobilised some resources to purchase drugs for the commencement of our work. That did not end there, one of the CHMC members who worked in a pharmaceutical company contacted some of the companies, and they provided us with the rest of the medicines we needed. Unlike other places, we do not send patients outside our facility to go and buy drugs. We have them here unless your condition is beyond us* (Midwife, CS 3).

Lastly, on access to healthcare services, implementing PPP was reported to significantly improve access to healthcare personnel, particularly community health nurses and midwives. Particularly in case sites 3, 5 and 6, we found that many newly posted nurses and nursing students on clinical internships in the communities were supported with free accommodation and sometimes free food from individual community members. This was noted as an important motivator to help retain such skilled health personnel in the communities:

*Most of my colleagues posted to other communities went for reposting because they did not have places to stay. These are remote communities already, so if the people did not adopt good approaches to help make our stay here comfortable, we would go back. You can see that despite this place being a very remote community, more nurses are working here. The reason is that none of us pays for our accommodation. The community provided these things for free, and they provide us with foodstuffs from their farms almost every week. Because of this, almost every nurse posted here chooses to stay* (Community Health Nurse, CS 5).

### Responsive healthcare system

Implementing various PPP accountability measures, such as strong ‘voice’ mechanisms, effective channels for complaints and the use of scorecards, was noted as effective in building a responsive healthcare system. For instance, implementing community scorecards and having strong complaints and feedback channels were key strategies that influenced healthcare workers to be more responsive towards the needs of the people. A member of the CHMC remarked:

*We have a good system here where the community members can easily provide feedback on the kind of care they received from the clinic. This also makes the workers sit up to do their job. We have had a cause to report one midwife here to the District Health Office, and the person was re-posted from our community. They know we don’t take negative feedback or complaints lightly, so they also try to make sure their work meets our demands* (CHMC member, CS 3).

Additionally, involving user communities in identifying their own healthcare needs or in priority-setting was key to building an effective healthcare system that was responsive to the needs of the people:

*Our healthcare plans are led by inputs from our patients. Patients’ inputs are the focus of our decisions, and it guides our strategic planning and, particularly, our annual programme of work. Every year before we start the planning process, I get the community representatives on our hospital board included in the process and also use the inputs from our durbars* *^2^*  *and make sure our plans reflect the community’s concerns. To be honest, it has really helped increase our attendance here. The one-sided health professional-led planning must be abandoned, especially in communities like ours* (Health Administrator 1).

Lastly, the participants shared specific examples of how the adoption of local knowledge in PPP activities led to building a healthcare system that was more responsive to patient needs. While the six community sites differed in culture, values and expectations, implementing PPP in consideration of these unique characteristics and local contextual factors led to a more responsive health system:

*This community has its own traditional ways of communication, and the people understand that language better. For instance, when the gong-gong [talking drum] is sounded, it means the traditional council is inviting the community for important information. It is compulsory for everyone in this community to be part of that meeting. People know that when the gong-gong is being sounded, then it is traditionally compulsory for everyone to pay attention to the announcement and honour it. So as a volunteer, [when] there are important issues, I mostly tell the health workers to work through the chiefs using these traditional means* (CHV, CS 3).

### Improved community–health worker relationships

Another important role of PPP as raised by participants was its ability to improve the working relationships between communities and healthcare workers. At the six community sites, strong bonds and good working relationships had been developed with their healthcare workers. This was primarily facilitated by the important coordinating role of the CHMCs and CHVs, particularly in reducing the power imbalances that had typically existed between communities and health workers. A Community Health Nurse explained:

*Community engagement has improved our working relationships with the community. It was not easy winning the community to our side. We thank the CHMC members and even the CHVs who have been very instrumental in breaking that power gap between health workers and the community. Now, the community walk to us freely to ask their important health questions or even other non-health-related issues* (Community Health Nurse, CS 1).

Participants also commented on the advantages of improved relationships between health workers and the community. Particularly, some participants noted that the CHMC members and the CHVs have helped in the quick resolution of conflicts between healthcare workers and the community, as well as addressing complaints from either side:

*Because there is a good relationship between our community and the health staff now, we try to resolve most misunderstandings quickly to ensure it does not progress into becoming something bad. At least, that has really helped the nurses not to harbour any bad feelings in their hearts about the community, and our community also does the same for the health staff. At least we have this friendly working environment, especially in all our health-related activities* (CHMC member & CHV, CS 4).

### Improved health-seeking behaviour

Participants observed PPP to have significantly improved the health-seeking behaviour of communities, mainly through the use of CHVs in frontline community service roles. CHVs are lay people selected from among the community to work closely with community health nurses or public health officers to promote quality healthcare services in their communities. Participants noted that the CHVs had the benefits of community knowledge and popularity, which helped them to identify or contact sick patients and other residents easily. They also assisted healthcare workers, particularly community health nurses, in knowing the best times and approaches to optimize community engagement. CHVs were said to have significantly helped change the community members’ attitudes towards staying home when sick and against self-medication. A Community Health Nurse elaborated:

*When we started this clinic here, our cases were less. Then our CHVs advised us to move into the communities. They took us around to identify people who were actually treating very bad wounds and other sicknesses with herbal medicines in their homes. The CHVs helped us talk to many of them until finally, many accepted to bring their cases to the clinic* (Community Health Nurse, CS 5).

PPP was reported to have also contributed to enhanced health-seeking behaviours in the communities. For instance, participants mentioned that in communities with difficulty accessing healthcare services, people resorted to self-treatment, including taking unprescribed herbal remedies, which eventually worsened their health conditions. A CHV narrated the helpless nature of their job pre-PPP, particularly when their advice to residents against herbal practices meant nothing to them because there was no easy access to health care services:

*We will go and talk to them about the need to seek proper healthcare services in hospitals when they are sick. After doing all the education, they ask you a simple question; how do we go to the hospital? Because before they get access to a health facility, they walked many hours or sometimes crossed dangerous rivers to access healthcare. So, their actions, though wrong, we could do nothing about it. Now, I can tell you that with the clinic here, this has very much improved. It is not common now to see many people going for herbal preparations from the farms to cure their diseases* (CHV, CS 6).

Moreover, the study also found that engagement facilitated by traditional leaders significantly contributed to improving the health-seeking behaviour of the communities. Notably, in some case sites, the Chiefs made pronouncements of punishing any community member who was involved in self-medication without seeking proper medical attention from the clinic. Due to the importance attached to the Chief’s word in these communities, participants confirmed this improving health-seeking behaviours:

*The landlord of the community, our … Chief, had made it an abomination for anyone to stay home and treat their sickness without going to the clinic. We all contributed to bringing the clinic here. We pay almost nothing for the service there, so why must you stay home and treat your disease? We don’t want it to become worse before we begin soliciting money to support the person. So, in this community, you have to go to the clinic when you are sick. It has now become part of our lifestyle* (Resident/Opinion leader, CS 6).

### Enhanced community empowerment

Effective implementation of PPP also empowered communities to take an active interest in various activities to improve the health system. Across all six community sites, participants reported that as PPP transformed the power relationships between communities and healthcare institutions, members of the community, particularly those from the minority ethnic groups, were empowered to participate in decision-making to improve the health system:

*Community engagement has improved people’s confidence to speak up, especially when they are not happy about healthcare delivery. Now, to some extent, we are also part of their decision-making. When we go to community durbars, you see everyone in this community, male, female, young, old etc., all have a common voice to participate in discussions which can eventually lead to improving our health activities here* (Assemblyman, CS 2).

In addition, the participants shared their experiences of how the implementation of various PPP accountability measures, such as the scorecard system (a quality indicator used in assessing gaps in health service delivery), empowered the community members:

*The scorecard system has really energised our engagement activities and also given so much confidence and power to our people to be part of decision-making. Now, with the scorecard system, we have the power to also report the health workers. That, in a way, has forced them to make us part of most of their decisions so that they gain our support. As CHMC member, this makes me feel empowered more about my work* (CHMC member & CHV, CS 1).

### Improvement in health outcomes

Finally, the findings of our study revealed that PPP activities improved health outcomes across all six community sites. Notably, all district directors interviewed mentioned various forms of improvements in health outcomes across several key health indicators following the implementation of PPP in the communities.

*In [Case Site], for instance, one of our biggest challenges was that they were over-reliant on herbalists because they did not have any health facility. Every year in our annual district health review, the community performed poorly, especially in all our major health indicators like maternal mortality, infant mortality, and even sending late cases to our district hospital. Now, after a few years of collaborating with the community to improve healthcare services there, including the community leading the construction of that clinic, they have overturned their story to be one of the best and a model community in this district* (District Director of Health Service 1).

Another District Director of Health Service shared a similar experience:

*We had most of our maternal mortalities coming from that community. We did all the major education and sent doctors there regularly. It worked, but just for a short while. We changed the approach to now join hands with the community in terms of engaging and collaborating together in many decisions to improve the situation, and that was what changed the situation. Allowing the community to own some of these things and we, the health workers supporting them with our expert advice and service is the best way to resolve most health challenges in the communities. As I have already mentioned, we cannot keep dictating to communities. It won’t work. Let them own their healthcare decisions* (District Director of Health Services 2).

Analyses of the interviews also revealed that PPP led to an overall improvement in outcomes across several key health indicators. For instance, in another case site, we found the community had positively improved from being among the worse for late reporting of Buruli Ulcer cases in the district to being among the best. The participants primarily credited this to an increased involvement of the Chief and the implementation of other PPP activities, such as the use of CHVs in ensuring cases were identified and reported early. An interview with the District Director of Health Service noted:

*When it comes to Buruli Ulcer reporting, … [Case Site] was among the worse communities which reported late to the hospital. You know how Buruli Ulcer works - if the case is detected late, you cause so much permanent disability to the person. Even though we implemented some approaches to dealing with this, I think our community engagement approaches and the role played by their Chief and the CHVs made a huge difference. Now, they are among our best-performing communities in the districts in terms of early diagnosis of Buruli Ulcer* (District Director of Health Services 3).

## Discussion

One key goal behind implementing PPP is to improve health and the overall health system. This study examined the experiences and perspectives of participants on how PPP functions across the Ghanaian health system and its role in health system improvement. Despite PPP being recognized in Ghana’s national health policy and legislated as a key strategy for improving the health system, we found that PPP was largely functional at only the community level rather than the entire health system. This limited PPP implementation across higher levels of the health system could potentially affect its overall role in the whole health system. The WHO’s Community Engagement Framework for People-Centred and Resilient Health Services (CEQ) identifies the strength of any health system as emanating from interconnections between different health system levels (Who, 2017). Therefore, the findings of this study provide important lessons for other health systems, particularly sub-Saharan African countries, to ensure having truly effective PPP by implementing it across all health system levels.

Regarding PPP’s role on health and health systems, participants perceived significant improvements following the implementation of PPP, albeit mainly at the community level. Firstly, PPP improved community members’ access to healthcare services. This is particularly important for resource-poor countries where access to healthcare services has been challenging. As noted by Atinga et al., drawing on such community-level support in lower-middle income countries (LMICs) will significantly complement government’s efforts to provide accessible healthcare services to the population (Atinga *et al*., 2019). For instance, our study found that through PPP, four communities were able to establish new healthcare facilities to improve their access to healthcare services. This was particularly crucial for these rural communities which faced major problems of access to health facilities (Sulemana and Dinye, 2014). We also observed from two communities that PPP had improved their access to medicines and diagnostic services. In these communities, before the PPP interventions, many patients avoided the inconvenience of travelling long distances from their communities for diagnostic services, particularly x-rays, laboratory investigations and prescribed medications. This had negative consequences on their treatment outcomes. Other studies conducted in Ghana have also reported improved access to healthcare services following the implementation of PPP (Baatiema *et al*., 2013; Alhassan *et al*., 2016; Yeboah and Jagri, 2016), although previous studies did not report improved access to medicines or diagnostic services as found in the present study.

PPP was also perceived by the participants to have improved the health system’s responsiveness to patient needs. A combination of PPP activities contributed to this, including allowing communities to identify their own healthcare needs; implementing effective accountability measures (including using the scorecard system, durbars and channels for making complaints); and using ‘local knowledge’ for the design and implementation of health activities in alignment with Campbell and Jovchelovitch’s social theory of participation (Campbell and Jovchelovitch, 2000). We noted that implementing key accountability measures prompted community healthcare workers to act more responsively towards patient needs. Although previous studies in Ghana had reported that the Ghanaian healthcare system was generally not responsive to the needs of patients (Agyei *et al*., 2020; Wu *et al*., 2021), our study findings showed that in communities where PPP was effectively implemented, the participants perceived the health system as responsive to their healthcare needs. Further, we found that involving communities in identifying their healthcare needs promoted a more responsive health system. As argued by Potts and Hunts, PPP, apart from being a fundamental right for patients or the public, also offers the healthcare system an opportunity to be generally responsive to the needs of the people it serves (Potts and Hunt, 2008). Our study found that PPP significantly improved the health worker–patient relationship by reducing the power gaps between health workers and the ordinary members of the community. Ghanaian society generally regards medical professionals, especially doctors and nurses, as having high status (Sakeah *et al*., 2021). For this reason, there has been a distance between ordinary community members and professional health workers. This situation may have contributed to community members preferring healthcare treatment from untrained local herbal practitioners rather than ‘elite’ healthcare workers (Peprah *et al*., 2018). However, this study found that the CHMCs and CHVs played significant roles in bridging this gap. Like most sub-Saharan African countries, Ghana has been identified as having poor health-seeking behaviours (Kuuire *et al*., 2016; Adedokun and Yaya, 2020). This is notably worse in rural communities where several factors influence people’s behaviour towards seeking healthcare services (Laar *et al*., 2013; Kuuire *et al*., 2016). In this study, PPP was found to have improved health-seeking behaviours especially using CHVs in frontline role for community health activities. This enabled health workers to employ the best local strategies to improve the community members’ understanding in making the right choices about their health rather than resorting to self-medication or seeking only traditional spiritual solutions to sickness. A study conducted in Bangladesh had similar findings, where the implementation of community-based partnership approaches was key to improving health-seeking behaviour in the communities (El Arifeen *et al*., 2013). Moreover, our findings showed that PPP greatly empowered the ordinary members of the community during decision-making in healthcare. As argued by Charles and DeMaio, empowering lay representatives such as CHVs and CHMCs ensures decision-making or policies reflect community values, preferences and lifestyles, which is key to improving health systems (Charles and Demaio, 1993). In this study, some key PPP activities, such as the accountability measures (including the various complaints mechanisms, scorecards, etc.), empowered community members to speak up and demand their rights. A similar study conducted in Estonia also found that the implementation of PPP programmes empowered community members to demand their rights and inclusion in key healthcare decision-making processes (Kasmel and Andersen, 2011).

Finally, our study participants across all communities indicated PPP is associated with an overall improvement in health outcomes. We noted that the involvement of CHVs contributed to early detection of diseases, therefore, avoiding a poor prognosis. For instance, in two communities, participants perceived that PPP had positively influenced the early detection of neglected tropical diseases such as Buruli Ulcer, Guinea worm infections and Onchocerciasis. This was largely due to using community members such as CHVs and the CHMCs for frontline roles, which improved people’s trust in the health system and supported adherence to treatment protocols to ensure successful outcomes. Previous studies in Ghana have similarly found that PPP activities also improved outcomes in malaria infections (Boakye *et al*., 2018) and lowered the incidence of HIV/AIDS infections (Aboagye-Sarfo *et al*., 2015).

Findings from this Ghanaian study also provide crucial lessons for other countries, especially those in sub-Saharan Africa. We note from our study that whilst it is important to involve communities in identifying their own healthcare needs, they must also be involved in the design and implementation of health activities as this greatly improves health and the overall health system. Again, the active involvement of CHVs in frontline role for community health activities is important to improve the health system. Overall, health systems must avoid tokenistic approaches to PPP by ensuring implementation is done across all health system levels.

Despite this study providing a strong understanding of PPP’s functioning in Ghana and its role in improving the health system, there are some limitations. Firstly, the views presented in this paper reflect only the personal experiences of participants from three selected districts of Ghana. As a result, our findings may not be representative of the views or experiences of PPP across all communities or districts in Ghana. Secondly, we purposefully selected case sites with high-functioning PPP initiatives. As a result, the findings may not be representative of PPP initiatives across the country. Notwithstanding, these study sites were carefully selected to ensure our study provided a detailed and contextualized understanding of PPP’s general functioning and role in the Ghanaian health system.

## Conclusion

Overall, we note that effective implementation of PPP is critical to improving health systems. Although Ghana has legislation and policies for promoting PPP implementation at various levels of the health system, implementation has so far been limited to only the community level. This, however, limits PPP’s role in health system improvement to only a few communities where PPP is functional. Thus, to maximize the role of PPP in health system improvement, all key health system actors must focus on implementing PPP across all health system levels. This includes actively involving CHVs in frontline role for community health activities as well as avoiding tokenistic approaches to PPP implementation across the various health system levels. Finally, we note that for many resource-poor countries, it is vital to prioritize PPP to complement governmental efforts to increase access to healthcare services; PPP supports patient-centred healthcare systems’ responsive to patient needs and improving overall health outcomes.

## Data Availability

The authors confirm that the data supporting the findings of this study have been reported within the article.

## Footnotes

1. Assembly members are locally elected representatives of the community or their electoral area to serve them at the District Assembly (local council).

2. Community durbars in the context of this study are large gatherings of people in a community called to discuss their health-related matters.

## Abbreviation

PPP = patient and public participationMOFEP = Ministry of Finance and Economic PlanningRDHS = Directorate of Health ServicesDDHS = District Directorate of Health ServicesCHAG = Christian Health Association of GhanaCHPS = community-based health planning and servicesMOH = Ministry of HealthCHMC = community health management committeesCHVs = community health volunteersCS = case site

## Reflexivity Statement

The authors are a mix of two males and one female of different levels of seniority—Junior, Early to Mid-career and Senior. Expertise spans health systems, public policy, patient–public engagement and qualitative research. Two authors have experience in lower and upper-middle-income countries, with one author having extensive public policy and health systems experience in high-income countries.
